# Phase angle by electrical bioimpedance is a predictive factor of hospitalisation, falls and mortality in patients with cirrhosis

**DOI:** 10.1038/s41598-021-99199-8

**Published:** 2021-10-14

**Authors:** Eva Román, Maria Poca, Gerard Amorós-Figueras, Javier Rosell-Ferrer, Cristina Gely, Juan C. Nieto, Silvia Vidal, Eulàlia Urgell, Andreu Ferrero-Gregori, Edilmar Alvarado-Tapias, Berta Cuyàs, Elvira Hernández, Rosalia Santesmases, Carlos Guarner, Àngels Escorsell, German Soriano

**Affiliations:** 1 Escola Universitària d’Infermeria EUI-Sant Pau, Barcelona, Spain; 2 Department of Gastroenterology, Hospital de la Santa Creu i Sant Pau, Mas Casanovas, 90, 08041 Barcelona, Spain; 3 Department of Cardiology, Hospital de la Santa Creu i Sant Pau, Barcelona, Spain; 4 Department of Biochemistry, Hospital de la Santa Creu i Sant Pau, Barcelona, Spain; 5 Department of Immunology, Hospital de la Santa Creu i Sant Pau, Barcelona, Spain; 6 Universitat Autònoma de Barcelona, Barcelona, Spain; 7 CIBERehd, Instituto de Salud Carlos III, Madrid, Spain; 8 CIBERCV, Instituto de Salud Carlos III, Madrid, Spain; 9 Department of Electronic Engineering, Universitat Politècnica de Catalunya, Barcelona, Spain; 10 Research Institute IIB-Sant Pau, Barcelona, Spain; 11 INCLIVA Health Research Institute, Valencia, Spain; 12 Foundation for the Promotion of Health and Biomedical Research of Valencia Region (FISABIO), Valencia, Spain

**Keywords:** Biomarkers, Diseases, Gastroenterology, Medical research

## Abstract

The phase angle is a versatile measurement to assess body composition, frailty and prognosis in patients with chronic diseases. In cirrhosis, patients often present alterations in body composition that are related to adverse outcomes. The phase angle could be useful to evaluate prognosis in these patients, but data are scarce. The aim was to analyse the prognostic value of the phase angle to predict clinically relevant events such as hospitalisation, falls, and mortality in patients with cirrhosis. Outpatients with cirrhosis were consecutively included and the phase angle was determined by electrical bioimpedance. Patients were prospectively followed to determine the incidence of hospitalisations, falls, and mortality. One hundred patients were included. Patients with phase angle ≤ 4.6° (n = 31) showed a higher probability of hospitalisation (35% vs 11%, p = 0.003), falls (41% vs 11%, p = 0.001) and mortality (26% vs 3%, p = 0.001) at 2-year follow-up than patients with PA > 4.6° (n = 69). In the multivariable analysis, the phase angle and MELD-Na were independent predictive factors of hospitalisation and mortality. Phase angle was the only predictive factor for falls. In conclusion, the phase angle showed to be a predictive marker for hospitalisation, falls, and mortality in outpatients with cirrhosis.

## Introduction

Patients with cirrhosis are predisposed to adverse outcomes, such as admissions to hospitals and long-term care centres, falls and fractures, and premature mortality^1–4^. These outcomes are related not only to the degree of liver insufficiency but also to frailty^2,5,6^. Recent years have seen growing interest in the frailty syndrome in association with chronic diseases, including cirrhosis, regardless of patients’ age^5–7^. Frailty in patients with cirrhosis is understood as a condition of weakness or lability due to physical and psychological deterioration related to the progression of the disease, the development of complications, and associated comorbidities^1,2,5,6^.

In addition to the adverse outcomes, other findings related to the frailty syndrome that are often present in patients with cirrhosis are functional and cognitive deterioration, impairment in health-related quality of life, immune system disturbances, and alterations in body composition^2,5,7–11^. Patients with cirrhosis frequently present alterations in body composition related to frailty and prognosis, such as sarcopenia or decreased muscle mass and/or function, decreased bone mass, and excess water and fat^12–15^.

Electrical bioimpedance is a simple, non-invasive, and innocuous method to estimate body composition. It is based on the passage of a weak, alternating, electrical current through the body in order to measure the voltage generated. The ratio of voltage to current is the impedance and can be expressed as a complex vector with two components: the electrical resistance (R) and the reactance (Xc). Resistance is linked to the opposition of the electrical current to flow through intra- and extracellular ionic solutions of muscle and fat, and the reactance is linked to the integrity of cell membranes and tissue interfaces. Alternatively, the impedance vector can be expressed as a phase angle (PA)^11,14,16–19^. Regression analysis has been used to derive empiric equations with the use of R and Xc together with anthropometric measurements, such as height and weight, to predict body compartment volumes^16^. The advantage of the PA is that it makes the measurement of any anthropometric parameter unnecessary and is a global indicator of the body composition. Measurement of the PA is fast and portable, and unlike imaging techniques such as CT scan, it does not involve ionizing radiation^11,14,20^. These characteristics allow it to be easily integrated into daily clinical practice^11^.

It has been suggested that the PA is a more precise tool than other nutritional, biochemical or anthropometric parameters to assess frailty and prognosis in patients with chronic diseases^11,18,21–23^. Its usefulness as a prognostic factor to predict encephalopathy episodes and mortality in patients with cirrhosis has been demonstrated in previous studies^11,14,19,24^.

The aim of our study was to analyse the prognostic value of the PA to predict clinically relevant events, such as hospitalisation, falls, and mortality in patients with cirrhosis.

## Results

### Characteristics of patients according to PA

**Figure 1:**
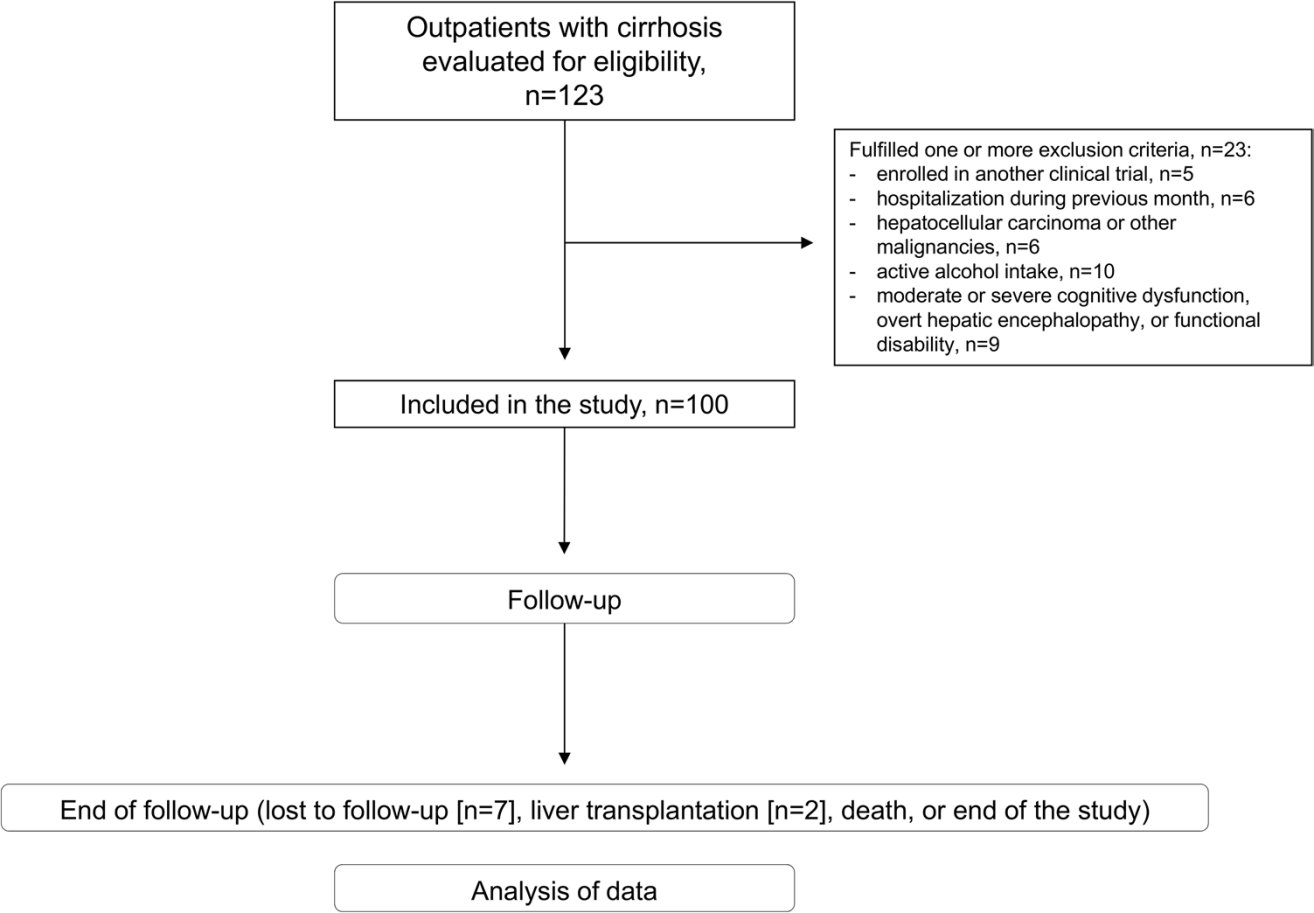
Flow-chart of the study.

Between February 2015 and January 2018 we evaluated 123 consecutive outpatients with cirrhosis. Twenty-three were excluded based on exclusion criteria. Characteristics of the remaining 100 patients are shown in the first column of Table 1. During a mean follow-up of 34.2 (14.8) months, two patients were referred to another centre to evaluate the possibility of liver transplantation and seven were lost to follow-up (Fig. 1). Considering the main outcomes, 30% of patients required hospitalisation, 23% presented falls, and 15% died. Using a ROC curve we found the best cut-off value for PA to predict mortality at 2-year follow-up was 4.6° (AUC 0.768, 95%CI 0.574–0.962, p = 0.008) with a sensitivity of 75% and specificity of 78%. Twenty-five per cent of patients were considered frail according to the Fried frailty criteria, and the best cut-off value to identify frail patients was also 4.6° (AUC 0.713, 95%CI 0.582–0.833, p = 0.001) with a sensitivity of 85% and specificity of 56%.

**Table 1:** Characteristics of patients: all patients, patients with phase angle (PA) ≤ 4.6° and patients with PA > 4.6°. Data are expressed as frequencies and percentages or mean (SD). P values in bold indicate statistical significance (p < 0.05).

	All patients (n = 100)	PA ≤ 4.6° (n = 31)	PA > 4.6ª (n = 69)	p (PA ≤ 4.6° vs. PA > 4.6°)
Age, years	63.8 (9.3)	67.4 (9.3)	62.2 (8.9)	**0.009**
**Gender, n (%)**				0.36
Male Female	68 (68) 32 (32)	19 (61.3) 12 (38.7)	49 (71) 20 (29.0)	
BMI^a^ (kg/m^2^)	27.4 (4.2)	28.3 (4.1)	27.0 (4.2)	0.16
**Aetiology, n (%)**				0.41
Alcohol Virus^b^ Alcohol + hepatitis C virus Other	63 (63) 14 (14) 7 (7) 16 (16)	17 (54.8) 7 (22.5) 2 (6.5) 5 (16.1)	46 (66.7) 7 (10.1) 5 (7.2) 11 (15.9)	
MELD-Na^c^ score	9.5 (3.0)	10.5 (3.4)	9.0 (2.7)	**0.02**
Child–Pugh class A/B/C, n (%)	81 (81)/18 (18)/1 (1)	20 (64.5)/10 (32.3)/1 (3.2)	61 (88.4)/8 (11.6)/0	**0.01**
**Previous decompensation, n (%)**	74 (74)	24 (77.4)	50 (72.5)	0.80
Ascites Encephalopathy Variceal bleeding Infection	63 (63) 12 (12) 31 (31) 26 (26)	20 (64.5) 5 (16.1) 8 (25.8) 9 (29.0)	43 (62.3) 7 (10.1) 23 (33.3) 17 (24.6)	1.00 0.51 0.49 0.63
Ascites, n (%)	9 (9)	5 (16.1)	4 (5.8)	0.13
Comorbidity^d^	0.7 (0.8)	0.81 (0.83)	0.59 (0.81)	0.16
Previous falls, n (%)	14 (14)	7 (22.6)	7 (10.1)	0.12
PHES^e^ score	− 0.65 (2.3)	− 1.19 (2.19)	− 0.41 (2.29)	0.11
Minimal hepatic encephalopathy^f^, n (%)	6 (6)	3 (9.7)	3 (4.3)	0.37
Beta-blockers, n (%)	49 (49)	18 (58.1)	31 (44.9)	0.28
Diuretics, n (%)	37 (37)	16 (51.6)	21 (30.4)	0.05
Non-absorbable disaccharides, n (%)	4 (4)	1 (3.2)	3 (4.3)	1.00
Antibiotics, n (%)	7 (7)	3 (9.7)	4 (5.8)	0.67
Bilirubin (µmol/L)	19.6 (11.0)	20.7 (13.1)	19.2 (10.0)	0.76
Albumin (g/L)	38.2 (4.8)	36.1 (5.2)	39.2 (4.3)	**0.003**
INR^g^	1.15 (0.18)	1.20 (0.22)	1.12 (0.15)	0.12
Serum sodium (mmol/L)	140.5 (3.0)	140.4 (2.8)	140.6 (3.4)	0.95
Serum creatinine (µmol/L)	76.2 (17.3)	83.1 (21.0)	73.1 (14.7)	**0.009**
Timed Up&Go test (sec)	10.2 (3.0)	11.2 (2.5)	9.8 (3.1)	**0.002**
Gait speed (m/sec)	1.06 (0.30)	0.95 (0.29)	1.11 (0.30)	**0.01**
Handgrip strength (kg)	25.3 (8.1)	22.0 (7.1)	26.9 (8.1)	**0.006**
Frail/Pre-frail/Robust^h^, n (%)	25 (25)/57 (57)/18 (18)	14 (45.2)/15 (48.4)/2 (6.5)	11 (15.9)/42 (60.9)/16 (23.2)	**0.004**

^a^BMI: body mass index.

^b^Hepatitis C virus: PA ≤ 4.6° five, PA > 4.6° four; Hepatitis B virus: PA ≤ 4.6° two, PA > 4.6° three.

^c^MELD-Na: Model for end-stage liver disease-sodium.

^d^Excluding cirrhosis from Charlson score.

^e^PHES: Psychometric hepatic encephalopathy score.

^f^PHES < -4.

^g^International normalized ratio.

^h^According to Fried frailty criteria: frail 3 criteria, pre-frail 1–2 criteria, robust 0 criteria.

As shown in Table 1, patients with a PA ≤ 4.6° (n = 31) were older and had more advanced liver insufficiency, lower serum albumin and more impaired renal function than patients with PA > 4.6° (n = 69). Moreover, patients with a PA ≤ 4.6° were more frail, as shown by the higher number of Fried frailty criteria, a higher Timed Up&Go test, slower gait speed, and lower handgrip strength. Regarding estimated body compartments, patients with a PA ≤ 4.6° showed higher extracellular water and lower muscle mass and body cell mass than patients with PA > 4.6° (Supplementary Table S1).

Potential biomarkers of frailty are shown in Table 2. Patients with a PA ≤ 4.6° showed lower levels of vitamin D and higher levels of cystatin C, TNF-α and IL-10 than patients with PA > 4.6°.

**Table 2:** Biomarkers in patients with phase angle (PA) ≤ 4.6° and patients with PA > 4.6°. Data are expressed as mean (SD). P values in bold indicate statistical significance (p < 0.05).

	PA ≤ 4.6° (n = 25)	PA > 4.6ª (n = 55)	p
Vitamin D (nmol/L)	13.7 (19.8)	22.7 (21.3)	**0.02**
Cystatin C (mg/L)	1.7 (0.6)	1.2 (0.3)	** < 0.001**
CRP (mg/L)	3.7 (2.5)	3.6 (5.5)	0.07
IL-6 (pg/mL)	20.4 (26.2)	14.2 (25.5)	0.13
TNF-α (pg/mL)	6.6 (13.7)	0.7 (3.2)	**0.008**
IL-10 (pg/mL)	313.7 (428.6)	118.3 (266.1)	**0.04**
Nitrites/nitrates (µmol/L)	49.4 (35.9)	44.2 (40.9)	0.44
Myostatin (ng/mL)	54.5 (52.2)	51.1 (37.8)	0.96
Testosterone (nmol/L) (n = 54 men)	17.8 (15.5) (n = 13)	22.0 (9.9) (n = 41)	0.25

*CRP* C-reactive protein, *IL-6* interleukin-6, *TNF-α* tumor necrosis factor-alpha, *IL-10* interleukin-10 .

The PA showed a positive correlation with handgrip strength (r = 0.40, p < 0.001) and serum albumin levels (r = 0.32, p = 0.001), and with testosterone concentration in men (r = 0.31, p = 0.02). However, it showed a negative correlation with age (r = −0.25, p = 0.01), the Fried frailty criteria (r = −0.38, p < 0.001), Child–Pugh score (r = −0.30, p = 0.002), MELD-Na score (r = −0.26, p = 0.008), cystatin C levels (r = −0.37, p = 0.001) and TNF-α concentration (r = −0.25, p = 0.02).

The characteristics of patients classified as frail or non-frail according to the Fried frailty criteria are shown in Supplementary Table S2. Frail patients showed a higher body mass index (probably suggesting sarcopenic obesity), a higher degree of comorbidity, more frequent previous falls and present ascites, and more impaired Timed Up&Go test, gait speed, handgrip strength and PA. However, there were no statistical differences between the two groups in age or in the degree of liver insufficiency as assessed by the Child–Pugh and MELD-Na scores. Regarding biomarkers, frail patients showed lower levels of vitamin D [12.4 (9.5) vs 23.7 (23.6) nmol/L, p = 0.04] and higher serum cystatin C [1.5 (0.5) vs 1.2 (0.3) mg/L, p = 0.005] than non-frail patients.

### Incidence of composite endpoint, hospitalisation, falls and mortality during follow-up according to PA

At 2-year follow-up (Table 3), patients with PA ≤ 4.6° had a higher incidence of the composite endpoint, hospitalisation and falls. They also had higher mortality throughout follow-up and at 2-year follow-up than patients with PA > 4.6°. Patients with PA ≤ 4.6° also needed more days in hospital, mainly due to complications of cirrhosis, and they presented a higher incidence of new episodes of ascites and hepatic encephalopathy. The incidence of variceal bleeding, infections or hepatocellular carcinoma, however, was similar in both groups. Supplementary Table S3 details the causes of hospitalisation at 2-year follow-up.

**Table 3:** Adverse outcomes during follow-up in patients with phase angle (PA) ≤ 4.6° and patients with PA > 4.6°.

	PA ≤ 4.6° (n = 31)	PA > 4.6ª (n = 69)	p
Composite endpoint^a^, n (%) Composite endpoint at 2 years, n (%)	17 (54.8) 16 (51.6)	25 (36.2) 11 (15.9)	0.12 ** < 0.001**
Hospitalisation, n (%) Hospitalisation at 2 years, n (%)	13 (41.9) 10 (35.5)	17 (24.6) 7 (10.1)	0.10 **0.01**
Days in hospital/year at 2 years Days in hospital due to complications of cirrhosis/year at 2 years	12.3 (30.0) 10.5 (28.9)	1.0 (5.4) 0.9 (5.4)	**0.003** **0.004**
**Complications of cirrhosis, n (%)**			
Ascites Encephalopathy Variceal bleeding Infections Hepatocellular carcinoma	8 (25.8) 5 (16.1) 1 (3.2) 3 (9.7) 1 (3.2)	6 (8.7) 1 (1.4) 2 (2.9) 7 (10.1) 5 (7.2)	**0.03** **0.01** 1.00 1.00 0.66
Long-term care centre admission, n (%) Long-term care centre admission at 2 years, n (%)	3 (9.7) 3 (9.7)	1 (1.4) 1 (1.4)	0.08 0.08
Falls, n (%) Falls at 2 years, n (%)	11 (35.5) 11 (35.5)	12 (17.4) 7 (10.1)	0.07 **0.004**
Deaths, n (%) Deaths at 2 years, n (%)	9 (29.0) 7 (22.6)	6 (8.7) 2 (2.9)	**0.01** **0.004**

Data are expressed as frequencies and percentages, or mean (SD). P values in bold indicate statistical significance (p < 0.05).

^a^Endpoint including hospitalisation, long-term care centre admission, falls, or death.

Regarding the injuries caused by falls, six patients with PA ≤ 4.6° had contusions, two had wounds and eight had fractures, while among patients with PA > 4.6°, five patients had contusions, one had a wound and seven had fractures (p = 0.06 vs patients with PA ≤ 4.6°). When considering healthcare needs due to falls, two patients with PA ≤ 4.6° were treated at a primary health care centre, three at the emergency room, and six were hospitalised. Among patients with PA > 4.6°, one patient was treated at a primary health care centre, four at an emergency room, and three required hospitalisation (p = 0.02 vs patients with PA ≤ 4.6°). Considering the whole series, comparing patients ≥ 65 years old (n = 48) and those < 65 years old (n = 52), we found that the incidence of falls at 2-year follow-up (20.8% vs 15.4%, respectively, p = 0.60) and throughout follow-up (25% vs 21.2%, p = 0.80) was only slightly higher in the former.

Mortality was higher in patients with a PA ≤ 4.6° than in those with PA > 4.6°. The causes of death in patients with PA ≤ 4.6° were cirrhosis-related in six patients (including one patient with hepatocellular carcinoma) and extrahepatic neoplasia in three patients. The causes of death in patients with PA > 4.6° were cirrhosis-related in five patients (including three patients with hepatocellular carcinoma) and acute myocardial infarction in one patient. Supplementary Table S3 details the causes of death at 2-year follow-up.

**Figure 2:**
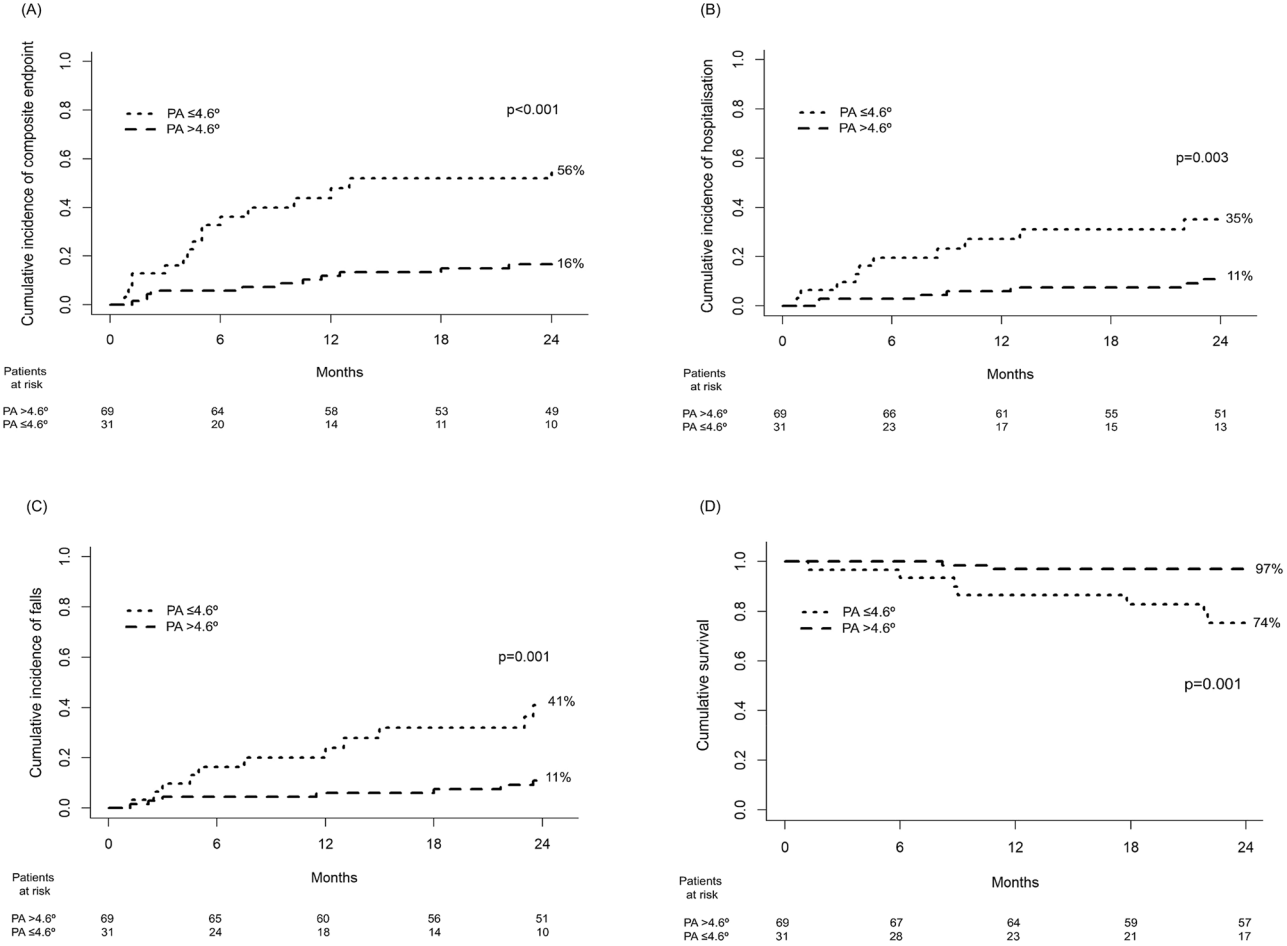
Probability of the composite endpoint (hospitalisation, admission to a long-term care centre, falls, or mortality) **(A)**, hospitalisation **(B)** and falls **(C)**, and survival **(D)** at 2-year follow-up in patients with phase angle (PA) ≤ 4.6° and patients with PA > 4.6°.

At 2-year follow-up, patients with PA ≤ 4.6° showed a higher probability of composite endpoint (56% vs 16%, p < 0.001), hospitalisation (35% vs 11%, p = 0.003) and falls (41% vs 11%, p = 0.001), and lower survival (74% vs 97%, p = 0.001) than patients with PA > 4.6° (Fig. 2).

### Multivariable analyses

Univariable and multivariable analyses are shown in Supplementary Table S4 and Table 4, respectively. Among all the factors that reached statistical significance in the univariable analysis, the PA ≤ 4.6° was the only independent predictive factor for the composite end-point at 2-year follow-up. The PA ≤ 4.6° and the MELD-Na score were the independent predictive factors of hospitalisation at 2-year follow-up. The PA ≤ 4.6° was the only predictive factor for falls, and PA ≤ 4.6° and the MELD-Na score were the independent predictive factors of mortality at 2-year follow-up. Due to a potential lack of statistical power an internal validation was made to test possible over-optimistic estimates in discriminative ability by bootstrap technique, and the results confirmed the predictive ability of PA.

**Table 4:** Multivariable analyses. Independent predictive factors for the composite endpoint, mortality, hospitalisation and falls at 2-year follow-up in all patients. P values in bold indicate statistical significance.

	HR (95%CI)	p
**Composite endpoint**^**a**^		
PA^b^ ≤ 4.6°	4.69 (2.16–10.18)	** < 0.001**
**Mortality**		
PA^b^ ≤ 4.6°	5.92 (1.20–29.16)	**0.03**
MELD-Na^c^	1.36 (1.11–1.67)	**0.003**

Competing risk analyses^d^		
	sHR (95%CI)	p
**Hospitalisation**		
PA^b^ ≤ 4.6°	3.31 (1.32–8.35)	**0.01**
MELD-Na^c^	1.20 (1.05–1.36)	**0.006**
**Falls**		
PA^b^ ≤ 4.6°	4.43 (1.72–11.40)	**0.002**

Discriminative ability for composite endpoint: estimated C-index 68.2%, after bootstrap 68.3%; for mortality: estimated C-index 88.4%, after bootstrap 85.7%; for hospitalisation: estimated C-index 72.8%, after bootstrap 72.1%; for falls: estimated C-index 67.4%, after bootstrap 67.8%.

^a^Endpoint including hospitalisation, long-term care centre admission, falls, or death.

^b^PA: phase angle.

^c^MELD-Na: model for end-stage liver disease-sodium.

^d^Death as competing event.

### Incidence of composite endpoint, hospitalisation, falls and mortality during follow-up according to MELD-Na and PA

**Figure 3:**
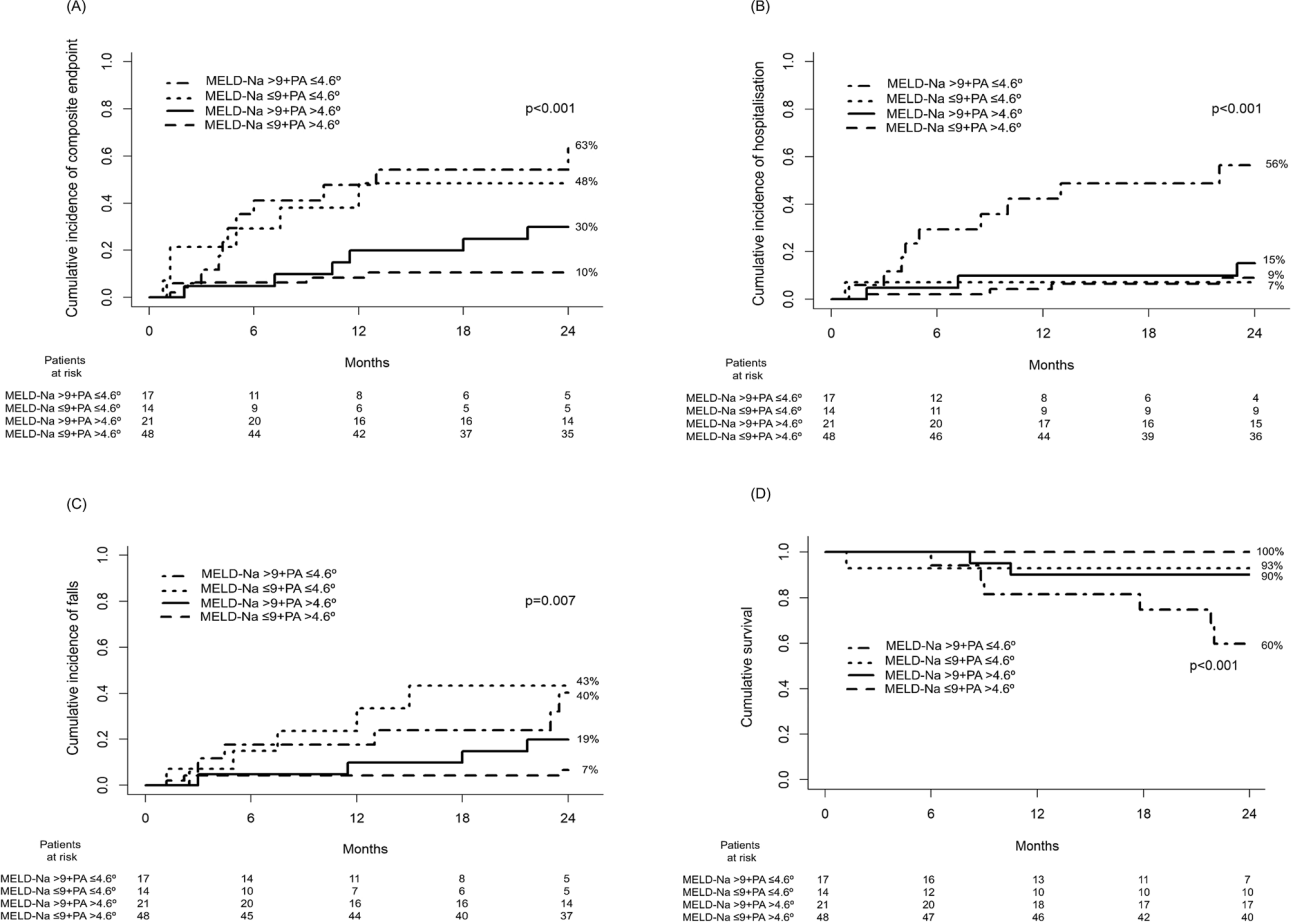
Probability of the composite endpoint (hospitalisation, admission to a long-term care centre, falls, or mortality) **(A)**, hospitalisation **(B)** and falls **(C)**, and survival **(D)** at 2-year follow-up classifying patients according to MELD-Na score ≤ or > 9 and phase angle (PA) ≤ or > 4.6°.

Figure 3 shows the probability of the composite endpoint, hospitalisation and falls, and survival classifying patients according to MELD-Na and PA. The cut-off for MELD-Na score was chosen according to the AUC of ROC curve for survival. As seen in this figure, the determination of the PA added predictive value to the MELD-Na score in several endpoints. This finding was confirmed by the results of the integrated discrimination improvement (IDI) and continuous net reclassification improvement (NRI) analyses (Supplementary Table S5).

## Discussion

The main finding in this study was the predictive value of the phase angle (PA) for hospitalisation, falls and mortality in outpatients with cirrhosis.

Determining the PA from the relationship between resistance and reactance by means of electrical bioimpedance allows a global assessment of body composition, nutrition and cell membranes integrity^11,16–20^. It therefore fits well with the holistic concept of frailty, a topic receiving increasing interest in patients with chronic diseases, including cirrhosis, as a prognostic factor and a target for therapeutic and prophylactic interventions^2,5–7^. In the present study patients with a low PA not only showed worse liver and renal function than those with a high PA, but also showed more impaired parameters of frailty according to indicators such as the Timed Up&Go test, gait speed, handgrip strength, and the Fried frailty criteria. The Fried frailty criteria have been widely used to evaluate frailty in several populations, including patients with cirrhosis, where they showed to be a predictive factor for hospitalisation, falls, and mortality^2,5,7^. However, Fried frailty criteria are time-consuming and non-specific for patients with cirrhosis. It is of note that since the present study was designed, faster and liver-specific indexes, particularly the Liver Frailty Index, have been developed and are now more commonly used than the Fried frailty criteria in this setting^1,5,6^.

Regarding biomarkers of frailty, patients with a low PA showed lower levels of vitamin D and higher levels of cystatin C, TNF-α and IL-10 than patients with a high PA. Vitamin D deficiency has been related to frailty and falls in the elderly^25^ and to frailty syndrome^2^ and mortality^26^ in patients with cirrhosis. An increase in cystatin C would reflect worse renal function and may be a predictive factor of mortality in these patients^27^. The increased TNF-α and IL-10 in patients with a low PA in comparison with those with a high PA suggests a more marked proinflammatory state in the former, and inflammation has been associated to frailty in the elderly^28^ and to prognosis in patients with cirrhosis^29^.

The PA has been shown to predict mortality in several populations, such as the elderly^30^ and patients with cancer^21^, chronic renal failure^22^ or heart failure^23^. In patients with cirrhosis, several authors have previously reported the value of PA to detect malnutrition^13^ and sarcopenia^11^, and as a predictive factor of hepatic encephalopathy^14,24^ or mortality^11,19,31^. However, the cut-off values used vary: 4.4° ^32^, 4.9° ^13,19^, 5.1° ^31^, 5.4° ^32^, 5.6° in males and 5.4° in females^11^. The probable explanation for these variations is the differences in the study populations and the different outcomes for which the cut-off was created. We used a cut-off of 4.6° because it gave the best area under the curve to predict mortality during follow-up in our population. Interestingly, 4.6° was also the best cut-off to identify frail patients according to Fried frailty criteria.

In the present study, besides mortality, we assessed other outcomes such as hospitalisations, admissions to a long-term care centre, and falls. These outcomes are relevant to patients with cirrhosis and they are also related to the frailty syndrome and sarcopenia^1,2,4–7,33–35^. Moreover, we used a composite endpoint including all these outcomes^2^.

We observed that patients with a low PA presented a higher incidence of the composite endpoint and—in agreement with previous studies—a higher mortality than patients with a high PA^11,19,31^. In addition, patients with a low PA also showed a higher incidence of hospitalisation and falls during follow-up when we evaluated these two outcomes separately. In the multivariable analysis, the PA was a relevant independent predictive factor of mortality, hospitalisation, and falls. This finding supports the value of a global quantitative evaluation of body composition using electrical bioimpedance in addition to the conventional scores assessing the degree of liver insufficiency, comorbidities and frailty, when determining prognosis in patients with cirrhosis^2,5,6,15^. Interestingly, PA improved the predictive value of the MELD-Na score to predict the composite endpoint, hospitalisation and mortality, and furthermore, PA was a predictive factor for falls while the MELD-Na score was not.

When analysing the different complications of cirrhosis during follow-up, we observed a higher incidence of ascites and hepatic encephalopathy in patients with PA ≤ 4.6° than in those with PA > 4.6°. This higher incidence of ascites in patients with a a low PA probably reflects a greater hyperhidration in these patients, as suggested by the increase in extracellular water estimated by electrical bioimpedance. Regarding hepatic encephalopathy, a lower PA would reflect a more sarcopenic status and therefore greater predisposition to this complication due to the difficulty in the clearance of ammonia by the muscle^9,24^. The decrease in muscle mass estimated by electrical bioimpedance and the lower handgrip strength observed in patients with PA ≤ 4.6° in comparison with patients with PA > 4.6° supports this hypothesis. We did not observe differences between the two groups regarding the incidence of variceal bleeding, infections or hepatocellular carcinoma, probably because these complications are less likely to be influenced by body composition.

Falls and fractures are a relevant adverse outcome in patients with cirrhosis^4,33,35^ and were more frequent in patients with a PA ≤ 4.6° than in those with a PA > 4.6°. Furthermore, fractures were a frequent cause of hospitalisation. Again, a more sarcopenic status in patients with a low PA could contribute to explaining these findings. In effect, patients with a low PA did worse in the Timed Up&Go test and gait speed, both tests evaluating the risk of falls. They also had a non-significant worse performance in the PHES than patients with a high PA that could also have contributed to the higher incidence of falls^4,33^. Considering the whole series, we found no statistical differences in the incidence of falls between patients ≥ 65 years old and those < 65 years old, and age was not a predictive factor for falls in the univariable analysis. This finding suggests that factors such as neurocognitive abnormalities and sarcopenia play a more relevant role than age in the predisposition to falling in patients with cirrhosis^33,35^.

Determining the PA by electrical bioimpedance has several advantages over other approaches to assess body composition or frailty, such as anthropometry, dual-energy X-ray absorptiometry, CT scan, Fried frailty criteria and other similar instruments. Electrical bioimpedance is simple, faster (it takes less than 5 min), less intrusive and more objective than anthropometry. Furthermore, it uses a portable, relatively non-expensive device and does not require radiation^11,14,20^.

Beyond the prognostic value of a single determination of the PA as observed in the present and other studies^11,19,32^, the sequential determination of the PA could be a simple and useful method to monitor the evolution of patients and the effect of interventions targeting frailty. In a recent longitudinal study, Lai et al^36^ reported that worsening or improvement of frailty—assessed by the Liver Frailty Index—affected prognosis independently of baseline frailty and MELD-Na. Regarding the effect of therapeutic interventions, it has been observed that the PA can change after therapeutic paracentesis^14^ or after treatment with a combination of nutritional therapy and supervised exercise^37^.

Our study has several limitations. First, the main limitation is the small sample size that precludes reliable subanalyses, such as by gender, age, previous decompensation of cirrhosis, or the presence of ascites, and limit the value of the multivariable analyses. Second, as commented earlier, PA can change during follow-up and we did not perform sequential evaluations. Aiming to minimize this limitation we focused our analysis on the first two years of follow-up. It would be interesting to evaluate the changes in the PA during follow-up and their prognostic significance. Third, although the incidence of adverse outcomes in our study was not negligible, particularly in patients with a low PA, the study population consisted of outpatients with well-preserved liver function. Therefore, our results can not necessarily be extrapolated to patients with more impaired liver function. Other authors, however, have demonstrated the prognostic value of the PA in patients with more advanced liver disease^19^. Fourth, estimating the different body compartments (i.e. extracellular body water and muscle mass) from reactance, resistance and PA using a monofrequency impedance meter in patients with cirrhosis is probably inaccurate and only valid as an approximation^11^. A multifrequency device could be more accurate to evaluate the specific body compartiments in these patients^14,17^. Finally, our study lacks a validation cohort. However, bootstrap analysis showed consistent results in internal validation. Further studies with a higher number of patients are necessary to validate the findings of the present study.

We conclude that the PA appears to be a simple and useful tool that can help to predict adverse outcomes such as hospitalisations, falls and mortality in outpatients with cirrhosis and preserved liver function. Identifying high-risk patients allows implementation of preventive strategies with the aim of improving survival and health-related quality of life.

## Patients and methods

### Design of study

This prospective observational study was performed in consecutive outpatients with cirrhosis visited at Hospital de la Santa Creu i Sant Pau, a tertiary care centre in Barcelona, Spain.

At inclusion to the study, we recorded demographic data and data regarding liver disease, including aetiology, degree of liver insufficiency and previous decompensations, and comorbidities. We assessed PA using electrical bioimpedance^11,30^, frailty^2,7^, risk of falls^33,34,38^, comorbidity^2,39^, cognitive function^40,41^, and potential biomarkers of frailty^2,25–28,42–44^. The incidence of hospitalisation, falls and mortality was prospectively evaluated and predictive factors were analyzed.

### Study participants

Participants were selected from among all consecutive outpatients with cirrhosis visited at the nursing outpatient clinic at Hospital de la Santa Creu i Sant Pau. The inclusion criteria were: patients aged ≥ 18 years diagnosed of liver cirrhosis by means of liver biopsy, or by clinical, analytical and ultrasonographic findings. The exclusion criteria were similar to those used by our group in previous studies^2,4,33^: age < 18 years, severe hepatic insufficiency (Model for end-stage liver disease [MELD] > 25), hospital admission in the previous month, hepatocellular carcinoma or any other active neoplastic disease, expected survival < 6 months, active alcohol intake (< 3 months), functional disability for activities of daily living (Barthel < 80%), moderate or severe cognitive dysfunction (Short portable mental status questionnaire [Pfeiffer test] ≥ 5 points), and overt hepatic encephalopathy.

### PA by electrical bioimpedance

Using the total body impedance meter BIA 101 at a signal frequency of 50 kHz and the software BodyGram TM 1.31 (Akern, Florence, Italy) we recorded the electrical resistance (R), reactance (Xc) and PA for each patient, and estimated body compartments after adjusting for age, sex, weight and height. Measurements were made in supine position with 4 conventional electrodes: 2 on the wrist and 2 on the ipsilateral foot. Indications before the test were no food or drink in the previous 4 h, no exercise in the previous 12 h, an empty bladder and removal of jewellery and clothes with metallic elements^11,30^.

### Fried frailty criteria

Frailty was defined on the basis of Fried’s five indicators from the Cardiovascular Health Study^2,7^: unintentional weight loss, reduced handgrip strength, slow gait speed, self-reported exhaustion, and low physical activity. The assessment of each criterion is detailed in Supplementary information.

### Risk of falls

The risk of falling was assessed using the Timed Up&Go test and gait speed^33,34^. The Timed Up&Go test measures the time the patient needs to stand up from a chair, walk 3 m, turn around, walk back and sit down again in the chair without support^33,38^. Gait speed was calculated according to the time taken to walk 5 m^34^.

### Comorbidity

The degree of comorbidity was assessed using a modified Charlson index^2,39^, excluding cirrhosis from the calculation (Supplementary information).

### Cognitive function

We assessed cognitive function using the Psychometric hepatic encephalopathy score (PHES) battery, which is widely used to assess cognitive function in patients with cirrhosis. This battery consists of the following neuropsychological tests: the Number Connection Test A and B, the Digit Symbol Test, the Serial Dotting Test and the Line Tracing Test^40,41^. Patients with a PHES score < − 4 points were considered to have minimal hepatic encephalopathy^41^.

### Potential biomarkers of frailty

At inclusion to the study, blood samples were stored at −80 °C for later determination of potential biomarkers of frailty, consisting of vitamin D, cystatin C, C-reactive protein, interleukin-6 (IL-6), tumour necrosis factor-alpha (TNF-α), interleukin-10 (IL-10), nitrites and nitrates, myostatin and testosterone in men^2,25–28,42–44^. C-reactive protein and cystatin C were measured by turbidimetric immunoassay (Architect Laboratories, USA), vitamin D was measured by liquid chromatography-tandem mass spectrometry (commercially available kit; Zivak Technologies, Turkey) and testosterone was measured by electrochemiluminescent immunoassay (cobas e601; Roche Diagnostics GmbH, Manheim, Germany). IL-6 (ImmunoTools, Friesoythe, Germany), TNF-α (BD Biosciences Pharmingen, San Diego, CA), IL-10 (BD Biosciences Pharmingen, San Diego, CA) and myostatin (CUSABIO Tech., Houston, TX, USA) were measured by ELISA, and nitrites and nitrates were measured using the Total Nitric Oxide and Nitrate/Nitrite Parameter Assay Kit (R&D Systems, Minneapolis, USA).

### Prospective evaluation of composite endpoint

We analyzed a composite endpoint that consisted of the main frailty-related outcomes in the general population: unexpected hospitalisation (we excluded planned hospitalizations for elective procedures), admission to a long-term care centre, falls, and mortality. These outcomes are especially relevant in patients with cirrhosis^2,4–7,33^. Patients were considered to present the composite endpoint when any of these four outcomes occurred.

### Prospective evaluation of incidence of falls during follow-up

Patients were prospectively evaluated through outpatient visits or phone calls every 3 months. During these evaluations we assessed the incidence of falls, the number of falls per patient, the severity of injuries, and the healthcare needed for falls, using a specific questionnaire^2,4,33^ (Supplementary information). Fall injuries were classified as contusion, wound or fracture. Healthcare needed was classified as primary care, emergency room care or hospitalisation^2,4,33^.

### Prospective evaluation of hospitalisations, admission to a long-term care centre and mortality during follow-up

Unexpected hospitalisations, admission to a long-term care centre, and mortality were prospectively assessed at each 3-monthly outpatient visit or phone call, and by review of medical records. We also determined the incidence of the main complications of cirrhosis during follow-up.

### Statistical analyses

Data are expressed as frequencies, percentages, and mean with SD. To compare categorical variables between two groups we used Fisher's exact test or the Chi^2^ test. To compare quantitative variables between two groups, we used the Student's "t" test if the variable presented a normal distribution and the Mann–Whitney test if it did not. The normality of the variables was assessed using the Kolmogorov–Smirnov test or the Shapiro–Wilk test. Correlations were assessed by the Pearson test or the Spearman test, according to data presented normal distribution or not, respectively.

We used the area under the Receiver Operating Characteristics (ROC) curves to determine the best cut-off value (maximising sensitivity and specificity) for a given parameter to predict an outcome.

Sample size was calculated according to previous data on mortality during follow-up in patients with cirrhosis and low PA (43%) and patients with high PA (16%)^13^. With 5% of patients lost during follow-up, an alpha error of 0.05, and a power of 0.80, 45 patients per group were necessary to show statistically significant differences in mortality during follow-up.

The probability of the composite endpoint or transplant-free survival was estimated by Kaplan–Meier curves and compared by log rank test. We used competing risk methods to estimate the probability of hospitalisation or falls during follow-up using the cumulative incidence function approach, in a context of competing risk analyses. The competing event was death during the study period. Patients who were submitted to another centre to evaluate the option of liver transplantation and those who were lost to follow-up were censored at the most recent visit to our center.

Variables with a p-value < 0.05 were included in the multivariable models. A backward elimination method was used to identify independent predictors of events. Factors predicting the composite endpoint or mortality were assessed by the Cox regression model and those predicting hospitalisation or falls were assessed by the Fine-Gray regression model in a competing risk scenario. The competing event for these analyses was death during the study period. The proportional hazard assumption was evaluated by the Schoenfeld residuals test. The discriminative ability of the models was assessed by the C-index. The internal validity of the final models was tested for 500 bootstrap resamples, using the “pec” package in the R Project for Statistical Computing. The integrated discrimination improvement (IDI) and continuous net reclassification improvement (NRI) indexes were implemented to quantify the added predictive value of PA^45^.

A 2-sided P value < 0.05 was considered statistically significant.

Statistical analyses were performed using the program released in 2017 IBM SPSS Statistics for Windows, Version 25.0; IBM Corp, Armonk, NY, United States; and R package (R Core Group, Version 3.6.1, 2019). Sample size was calculated using the computer program GRANMO version 7.12, released in 2012 and developed by the Institut Municipal d'Investigació Mèdica, Barcelona, Spain.

### Ethics declaration

The study conformed to the 1975 Declaration of Helsinki and Guidelines for Good Clinical Practice and was approved by the Clinical Research Ethics Committee at our institution (Comitè Ètic d’Investigació Clínica [CEIC] at Hospital de la Santa Creu i Sant Pau). All patients received information regarding their participation in the study and signed an informed consent form.

## Supplementary Information

Supplementary Information.

## Data Availability

The datasets generated during and/or analysed during the current study are available from the corresponding author on reasonable request.

## References

[1] Tandon P (2016). A rapid bedside screen to predict unplanned hospitalization and death in outpatients with cirrhosis: A prospective evaluation of the Clinical Frailty Scale. Am. J. Gastroenterol..

[2] Román E (2021). Frailty in outpatients with cirrhosis: A prospective observational study. Liver Int..

[3] Morales BP (2017). Early hospital readmission in decompensated cirrhosis: Incidence, impact on mortality, and predictive factors. Dig Liver Dis..

[4] Román E (2011). Minimal hepatic encephalopathy is associated with falls. Am. J. Gastroenterol..

[5] Kok B, Tandon P (2018). Frailty in patients with cirrhosis. Curr. Treat Options Gastroenterol..

[6] Lai JC (2019). Frailty associated with waitlist mortality independent of ascites and hepatic encephalopathy in a multicenter study. Gastroenterology.

[7] Fried LP (2001). Frailty in older adults: Evidence for a phenotype. J. Gerontol. A Biol. Sci. Med. Sci..

[8] Román E (2013). Falls and cognitive dysfunction impair health-related quality of life in patients with cirrhosis. Eur. J. Gastroenterol. Hepatol..

[9] Córdoba J, Minguez B (2008). Hepatic encephalopathy. Semin. Liver Dis..

[10] Montano-Loza AJ (2012). Muscle wasting is associated with mortality in patients with cirrhosis. Clin. Gastroenterol. Hepatol..

[11] Ruiz-Margáin A (2020). Phase angle from bioelectrical impedance for the assessment of sarcopenia in cirrhosis with or without ascites. Clin. Gastroenterol. Hepatol..

[12] Montano-Loza AJ (2016). Sarcopenic obesity and myosteatosis are associated with higher mortality in patients with cirrhosis. J. Cachexia Sarcopenia Muscle..

[13] Ruiz-Margáin A (2015). Malnutrition assessed through phase angle and its relation to prognosis in patients with compensated liver cirrhosis: A prospective cohort study. Dig. Liver Dis..

[14] Ontanilla-Clavijo G, Ampuero J, Borreguero S, Rosell J, Romero-Gómez M (2019). Usefulness of bioelectrical impedance analysis for monitoring patients with refractory ascites. Rev. Esp. Enferm. Dig..

[15] Tapper EB (2019). Body composition predicts mortality and decompensation in compensated cirrhosis patients: A prospective cohort study. JHEP Rep..

[16] Piccoli A, Pillon L, Dumler F (2002). Impedance vector distribution by sex, race, body mass index, and age in the United States: Standard reference intervals as bivariate Z scores. Nutrition.

[17] Kyle UG (2004). Bioelectrical impedance analysis—part I: Review of principles and methods. Clin. Nutr..

[18] Kyle UG, Genton L, Pichard C (2013). Low phase angle determined by bioelectrical impedance analysis is associated with malnutrition and nutritional risk at hospital admission. Clin. Nutr..

[19] Belarmino G (2017). Phase angle obtained by bioelectrical impedance analysis independently predicts mortality in patients with cirrhosis. World J. Hepatol..

[20] Molfino A, Johnson S, Medici V (2017). The challenges of nutritional assessment in cirrhosis. Curr. Nutr. Rep..

[21] Norman K, Wirth R, Neubauer M, Eckardt R, Stobäus N (2015). The bioimpedance phase angle predicts low muscle strength, impaired quality of life, and increased mortality in old patients with cancer. J. Am. Med. Dir. Assoc..

[22] Mushnick R (2003). Relationship of bioelectrical impedance parameters to nutrition and survival in peritoneal dyalisis patients. Kidney Int..

[23] Colín-Ramírez E (2012). Bioelectrical impedance phase angle as a prognostic marker in chronic heart failure. Nutrition.

[24] Ruiz-Margáin A (2016). Low phase angle is associated with the development of hepatic encephalopathy in patients with cirrhosis. World J. Gastroenterol..

[25] Neelemaat F (2012). Vitamin D decreases falls in malnourished older adults. J. Am. Geriatr. Soc..

[26] Finkelmeier F (2015). Low 25-hydroxyvitamin D levels are associated with infections and mortality in patients with cirrhosis. PLoS ONE.

[27] Jo SK (2019). Role of biomarkers as predictors of acute kidney injury and mortality in decompensated cirrhosis. Sci. Rep..

[28] Payette H (2003). Insulin-like growth factor-I and interleukin-6 predict sarcopenian very old community-living men and women: The Framingham Study. J. Am. Geriatr. Soc..

[29] Arroyo V (2021). The systemic inflammation hypothesis: Towards a new paradigm of acute decompensation and multiorgan failure in cirrhosis. J. Hepatol..

[30] Wilhelm-Leen ER, Hall YN, Horwitz RI, Chertow GM (2014). Phase angle, frailty and mortality in older adults. J. Gen. Intern. Med..

[31] Pagano AP (2020). Phase angle as a severity indicator for liver diseases. Nutrition.

[32] Selberg O, Selberg D (2002). Norms and correlates of bioimpedance phase angle in healthy human subjects, hospitalized patients, and patients with liver cirrhosis. Eur. J. Appl. Physiol..

[33] Soriano G (2012). Cognitive dysfunction in cirrhosis is associated with falls: A prospective study. Hepatology.

[34] Dunn MA (2016). Frailty as tested by gait speed is an independent risk factor for cirrhosis complication that require hospitalization. Am. J. Gastroenterol..

[35] Ezaz G, Murphy SL, Mellinger J, Tapper EB (2018). Increased morbidity and mortality associated with falls among patients with cirrhosis. Am. J. Med..

[36] Lai JC (2020). Changes in frailty are associated with waitlist mortality in patients with cirrhosis. J. Hepatol..

[37] Macías-Rodríguez RU (2016). Changes in hepatic venous pressure gradient induced by physical exercise in cirrhosis: Results of a pilot randomized open clinical trial. Clin. Transl. Gastroenterol..

[38] Viccaro LJ, Perera S, Studenski S (2011). Is timed up and go better than gait speed in predicting health, function, and falls in older adults?. J. Am. Geriatr. Soc..

[39] Charlson ME, Pompei P, Ales KL (1987). A new method of classifying prognostic comorbidity in longitudinal studies: Development and validation. J. Chronic Dis..

[40] Weissenborn K, Ennen JC, Schomerus H, Rückert N, Hecker H (2001). Neuropsychological characterization of hepatic encephlopathy. J. Hepatol..

[41] Romero-Gómez M (2006). Normality tables in the Spanish population for psychometric tests used in the diagnosis of minimal hepatic encephalopathy. Med. Clin. (Barc)..

[42] Serviddio G (2009). Frailty syndrome is associated with altered circulating redox balance and increased markers of oxidative stress. Int. J. Immunopathol. Pharmacol..

[43] McKay BR, Ogborn DI, Bellamy LM, Tarnopolsky MA, Pariseet G (2012). Myostatin is associated with age-related human muscle stem cell dysfunction. FASEB J..

[44] Sinclair M, Grossmann M, Hoermann R, Angus PW, Gow PJ (2016). Testosterone therapy increases muscle mass in men with cirrhosis and low testosterone: A randomised controlled trial. J. Hepatol..

[45] Pencina MJ, D'Agostino RB, D'Agostino RB, Vasan RS (2008). Evaluating the added predictive ability of a new marker: From area under the ROC curve to reclassification and beyond. Stat. Med..

