# Supplementary material for: Phase angle by electrical bioimpedance is a predictive factor of hospitalisation, falls and mortality in patients with cirrhosis
Source: Sci Rep. 2021 Oct 14;11:20415. doi: 10.1038/s41598-021-99199-8 (PMC8516941; doi:10.1038/s41598-021-99199-8)
Supplement: Supplementary file 1 — Supplementary Information. [file 41598_2021_99199_MOESM1_ESM.docx]

**Supplementary information**

**Title**

Phase angle by electrical bioimpedance is a predictive factor of hospitalisation, falls and mortality in patients with cirrhosis

**Authors**

Eva Román, Maria Poca, Gerard Amorós-Figueras, Javier Rosell-Ferrer, Cristina Gely, Juan C. Nieto, Silvia Vidal, Eulàlia Urgell, Andreu Ferrero-Gregori, Edilmar Alvarado-Tapias, Berta Cuyàs, Elvira Hernández, Rosalia Santesmases, Carlos Guarner, Àngels Escorsell and German Soriano

**Supplementary methods**

**Fried frailty criteria**

[Modified from Fried, L.P. *et al*. *J Gerontol A Biol Sci Med Sci.* **56**, M146-M156 (2001)]

Frailty was defined on the basis of the five Fried frailty criteria of the Cardiovascular Health Study:^1,2^ unintentional weight loss, reduced handgrip strength, slow gait speed, self-reported exhaustion, and low physical activity.

Patients were considered frail if they met ≥3 criteria, and non-frail if they met 0-2 criteria (including pre-frail 1-2 criteria, and robust 0 criteria).^1^

- Unintentional weight loss was considered a criterion of frailty when it reached 5 kg or 5% of body weight in the previous year.^1^
- Handgrip strength was measured in the dominant hand with a dynamometer (KERN MAP-BA-s-0910). The participant was seated comfortably with the arm bent at the elbow in a 90º angle. Instructions were given to grip the dynamometer with the maximum strength. We calculated the mean of three consecutive tests. Handgrip strength was considered indicative of frailty when it was below the lowest quintile of reference values adjusting for gender and body mass index.^1^

Cut-off for handgrip strength (kg) to be considered criterion for frailty:

Men BMI ≤ 24 ≤ 29 kg

BMI 24.1-26 ≤ 30 kg

BMI 26.1-28 ≤ 30 kg

BMI > 28 ≤ 32 kg

Women BMI ≤ 23 ≤ 17 kg

BMI 23.1-26 ≤ 17.3 kg

BMI 26.1-29 ≤ 18 kg

BMI > 29 ≤ 21 kg

- Gait speed: patients were considered frail for this criterion when they walked at ≤ 0.8 m/sec in a distance of 5 meters.^3-5^
- Self-reported exhaustion. In response to the question “How often in the last week did you feel this way?” patients were asked to respond to two statements from the Centre for Epidemiologic Studies Depression Scale (CES-D):

1) “I felt that everything I did was an effort”, and

2) “I could not get going”.

Patients were offered 4 possible answers: 0 = rarely or less than once day, 1 = a little or some of the time (1-2 days), 2 = often (3-4 days), or 3 = most of the time (5-7 days).

Patients who answered “2” or “3” to either of the two questions were considered frail for this criterion.^1^

- Physical activity was evaluated according to the short Spanish version of the International Physical Activity Questionnaire (IPAQ) ([www.ipaq.ki.se](http://www.ipaq.ki.se)).^6,7^ Patients were considered frail for this criterion if they scored below level 2 (moderate exercise, i.e. walking less than 30 minutes per day, 5 days a week).^7^

**References**

1. Fried, L.P. *et al.* Frailty in older adults: evidence for a phenotype. *J Gerontol A Biol Sci Med Sci.* **56**, M146-M156 (2001).
2. Román, E. *et al*. Frailty in outpatients with cirrhosis: a prospective observational study. *Liver Int.* **41**, 357-368 (2021).
3. Castell, M.V. *et al*. Frailty prevalence and slow walking speed in persons age 65 and older: implications for primary care. *BMC Family Practice.* **14**, 86 (2013).
4. Cruz-Jentoft, A.J. *et al*. Sarcopenia: European consensus on definition and diagnosis. Report of the European Working Group on Sarcopenia in Older People. *Age Ageing.* **39**, 412–423 (2010).
5. Cruz-Jentoft, A.J. *et al.* Sarcopenia: revised European consensus on definition and diagnosis. *Age Ageing.* **48**, 16-31 (2019).
6. Craig, C.L. *et al*. International physical activity questionnaire: 12-country reliability and validity. *Med Sci Sports Exerc.* **35**, 1381-1395 (2003).
7. Román Viñas, B., Ribas Barba, L., Ngo, J. & Serra Majem, L. Validity of the international physical activity questionnaire in the Catalan population (Spain). *Gac Sanit* . **27**, 254-257 (2013).

**Modified Charlson comorbidity index**

[Modified from Charlson, M.E. *et al*. *J Chronic Dis.* **40**, 373-383 (1987), excluding liver disease]

AIDS 6

Metastatic solid tumour 6

Any other tumour 2

Leukaemia 2

Lymphoma 2

Diabetes (with complications) 2

Moderate or severe renal disease 2

Hemiplegia 2

Diabetes (no complications) 1

Peptic ulcer disease 1

Connective tissue disease 1

Chronic pulmonary disease 1

Dementia 1

Cerebrovascular disease 1

Peripheral vascular disease 1

Congestive heart failure 1

Acute myocardial infarction 1

**Falls questionnaire**

[Román, E. *et al*. *Am J Gastroenterol.* **106**, 476-482 (2011)]

1. Have you had a fall in the last … months?

Yes No

1. If so, how many falls have you had during this period?
2. If you remember, please specify the dates and places:

- Falls at home
- Falls elsewhere

1. Did you hurt yourself in any of these falls?

Yes No

1. If so, please specify the type of injury-ies:

- Contusion
- Wound
- Fracture Type of fracture:

1. Did you seek medical care after your fall-s?

Yes No

1. If so, please specify the type of medical care received for each fall:

- Attended at a primary health centre
- Attended at a hospital emergency room
- Admitted to hospital

1. If admitted to another centre, can you provide a clinical report?

Yes No

1. If you were admitted to hospital, how many days were you there?
2. Did you have complications of your liver disease after the fall?

Yes No

1. If so, please specify which complications.

**Supplementary results**

Supplementary Table S1. Electrical bioimpedance results (resistance [R], reactance [Xc] and impedance [phase angle, PA]) and estimated body compartments in all patients, patients with PA ≤4.6º and patients with PA >4.6º. Data are expressed as mean (SD). P values in bold indicate statistical significance (p<0.05).

|  | **All patients**  **(n=100)** | **PA ≤4.6º**  **(n=31)** | **PA >4.6ª**  **(n=69)** | **p**  **(PA ≤4.6º vs**  **PA >4.6º)** |
| --- | --- | --- | --- | --- |
| Resistance (R) (Ohm) | 447.1 (70.4) | 435.2 (64.5) | 452.4 (72.8) | 0.26 |
| Reactance (Xc) (Ohm) | 41.4 (11.6) | 31.3 (7.0) | 45.9 (10.4) | **<0.001** |
| Impedance (PA) (º) | 5.40 (1.57) | 4.03 (0.58) | 6.02 (1.49) | **<0.001** |
| Total body water (L) | 41.6 (9.2) | 42.5 (10.0) | 41.1 (8.9) | 0.50 |
| Extracellular water (L) | 21.2 (5.3) | 23.9 (6.2) | 20.0 (4.4) | **0.002** |
| Intracellular water (L) | 21.7 (5.8) | 19.6 (6.6) | 22.6 (5.2) | **0.003** |
| Fat free mass (kg) | 48.6 (14.1) | 49.2 (17.7) | 48.4 (12.3) | 0.80 |
| Muscle mass (kg) | 40.3 (13.9) | 35.9 (13.7) | 42.3 (13.5) | **0.01** |
| Fat mass (kg) | 22.5 (12.3) | 25.1 (14.1) | 21.4 (11.4) | 0.25 |
| Body cell mass (kg) | 29.8 (11.4) | 26.5 (12.1) | 31.3 (10.8) | **0.002** |

Supplementary Table S2Final del formulario. Characteristics of patients: all patients, frail patients (according to Fried frailty criteria) and non-frail patients. Data are expressed as frequencies and percentages or mean (SD). P values in bold indicate statistical significance (p<0.05).

|  | **All patients**  **(n=100)** | **Frail patients**  **(n=25)** | **Non-frail patients**  **(n=75)** | **p**  **(Frail vs non-frail patients)** |
| --- | --- | --- | --- | --- |
| Age, years | 63.8 (9.3) | 65.0 (10.0) | 63.4 (9.1) | 0.50 |
| Gender, n (%)  - Male  - Female | 68 (68)  32 (32) | 16 (64)  9 (36) | 52 (69)  23 (31) | 0.63 |
| BMI^a^ (kg/m^2^) | 27.4 (4.2) | 29.4 (4.6) | 26.7 (3.9) | **0.01** |
| Aetiology, n (%)  - Alcohol  - Virus^b^  - Alcohol +  hepatitis C virus  - Other | 63 (63)  14 (14)  7 (7)  16 (16) | 15 (60)  3 (12)  3 (12)  4 (16) | 48 (64)  11 (14.6)  4 (5.3)  12 (16) | **0.72** |
| MELD-Na^c^ score | 9.5 (3.0) | 10.0 (3.25) | 9.3 (2.3) | 0.34 |
| Child-Pugh class  A/B/C, n (%) | 81 (81)/18 (18)/  1 (1) | 17 (68)/ 8 (32)/  0 (0) | 64 (85)/ 10 (13)/  1 (1) | 0.08 |
| Previous decompensation,  n (%)  - Ascites  - Encephalopathy  - Variceal bleeding  - Infection | 74 (74)  63 (63)  12 (12)  31 (31)  26 (26) | 20 (80)  20 (80)  6 (24)  7 (28)  11 (44) | 54 (72)  43 (57)  6 (8)  24 (32)  15 (20) | 0.60  0.06  0.07  0.81  **0.03** |
| Ascites, n (%) | 9 (9) | 5 (20) | 4 (5) | **0.04** |
| Comorbidity^d^ | 0.7 (0.8) | 1.1 (0.9) | 0.5 (0.7) | **0.002** |
| Previous falls, n (%) | 14 (14) | 7 (28) | 7 (9) | **0.04** |
| PHES^e^ score | -0.6 (2.3) | -0.9 (2.3) | -0.5 (2.2) | 0.57 |
| Minimal hepatic encephalopathy^f^, n (%) | 6 (6) | 2 (8) | 4 (5) | 0.64 |
| Beta-blockers, n (%) | 49 (49) | 14 (56) | 35 (47) | 0.49 |
| Diuretics, n (%) | 37 (37) | 12 (48) | 25 (33) | 0.23 |
| Non-absorbable  disaccharides, n (%) | 4 (4) | 1 (4) | 3 (4) | 1.00 |
| Antibiotics, n (%) | 7 (7) | 3 (12) | 4 (5) | 0.36 |
| Bilirubin (µmol/L) | 19.6 (11.0) | 16.7 (7.8) | 20.6 (11.8) | 0.16 |
| Albumin (g/L) | 38.2 (4.8) | 37.4 (5.5) | 38.6 (4.6) | 0.34 |
| INR^g^ | 1.15 (0.18) | 1.19 (0.22) | 1.13 (0.17) | 0.26 |
| Serum sodium (mmol/L) | 140.5 (3.0) | 140.8 (3.4) | 140.36(2.9) | 0.56 |
| Serum creatinine (µmol/L) | 76.2 (17.3) | 81.7 (22.5) | 74.3 (15.0) | 0.13 |
| Timed Up&Go test (sec) | 10.2 (3.0) | 12.3 (4.7) | 9.5 (1.8) | **<0.001** |
| Gait speed (m/sec) | 1.06 (0.30) | 0.89 (0.30) | 1.12 (0.29) | **<0.001** |
| Handgrip strength (kg) | 25.3 (8.1) | 21.0 (5.9) | 26.8 (8.3) | **<0.001** |
| PA^h^ (º) | 5.4 (1.5) | 4.6 (1.3) | 5.6 (1.6) | **0.001** |

^a^BMI: body mass index; ^b^Hepatitis C virus: Frail two, Non-frail seven, Hepatitis B virus:

frail one, Non-frail four; ^c^MELD-Na: Model for end-stage liver disease-sodium; ^d^Excluding cirrhosis from Charlson score; ^e^PHES: Psychometric hepatic encephalopathy score; ^f^PHES<-4; ^g^International normalized ratio; ^h^PA: phase angle.

Supplementary Table S3. Causes of the first hospitalisation and death at 2-year follow-up in patients with PA ≤4.6º and patients with PA >4.6º. Data are expressed as number of patients. ACLF: acute-on-chronic lver failure.

|  | **PA ≤4.6º**  **(n=31)** | **PA >4.6ª**  **(n=69)** |
| --- | --- | --- |
| Cause of hospitalisation | 2 encephalopathy  1 pneumonia +  encephalopathy +  ascites  1 variceal bleeding  1 respiratory  insufficiency  1 urinary infection  1 rib fractures after a fall + ascites  1 atrioventricular block  1 hearth failure  1 brain haemorrhage | 3 ascites  1 respiratory infection  1 respiratory infection + renal failure  1 cholecystitis  1 choledocholithiasis |
| Cause of death | 2 ACLF due to spontaneous  bacterial peritonitis  1 ACLF (including hepatorenal  syndrome) due to variceal  bleeding + infection (pneumonia  + bacteraemia)  1 ACLF due to respiratory infection  1 pancreatic cancer  1 lymphoma  1 lung oat-cell carcinoma | 1 hepatocellular carcinoma  1 ACLF after surgery for pancreatic  cyst |

Supplementary Table S4. Univariable analysis. All the statistically significant factors associated with the composite endpoint, mortality, hospitalisation and falls at 2-year follow-up in all patients. P values in bold indicate statistical significance.

|  | **HR (95%CI)** | **p** |
| --- | --- | --- |
| **Composite endpoint^a^** | | |
| MELD-Na^b^ | 1.15 (1.03-1.28) | **0.01** |
| Comorbitity (modified Charlson comorbidity index) | 1.50 (1.03-2.19) | **0.03** |
| Frailty (robust/pre-frail/frail according to Fried frailty criteria)^c^ | 2.31 (1.25-4.24) | **0.007** |
| PA^d^ ≤4.6º | 4.69 (2.16-10.18) | **<0.001** |
| Antidepressants | 3.58 (1.24-10.38) | **0.02** |
| Serum albumin (g/L) | 0.90 (0.84-0.97) | **0.007** |
| Cystatin C (mg/L) (n=80) | 2.55 (1.39-4.69) | **0.003** |
| Testosterone (nmol/L) (n=54 men) | 0.92 (0.87-0.98) | **0.01** |
| **Mortality** | | |
| MELD-Na^b^ | 1.42 (1.17-1.71) | **<0.001** |
| PA^d^ ≤4.6º | 9.19 (1.91-44.31) | **0.006** |
| Serum sodium (mmol/L) | 0.74 (0.63-0.86) | **<0.001** |
| Serum albumin (g/L) | 0.82 (0.72-0.92) | **0.002** |
| Cystatin C (mg/L) (n=80) | 3.84 (1.65-8.93) | **0.002** |
| TNF-α (pg/mL) (n=80) | 1.64 (1.11-2.42) | **0.01** |
| **Hospitalisation** | | |
| MELD-Na^b^ | 1.23 (1.09-1.38) | **<0.001** |
| Child-Pugh class | 3.00 (1.29-6.97) | **0.01** |
| Comorbitity (modified Charlson comorbidity index) | 1.77 (1.15-2.72) | **0.01** |
| Frailty (robust/pre-frail/frail according to Fried frailty criteria)^c^ | 3.29 (1.45-7.47) | **0.005** |
| Timed Up&Go test (sec) | 1.10 (1.05-1.16) | **<0.001** |
| Handgrip strength (kg) | 0.94 (0.90-0.98) | **0.006** |
| PA^d^ ≤4.6º | 3.98 (1.54-10.30) | **0.004** |
| Diuretics | 2.96 (1.15-7.59) | **0.02** |
| Serum albumin (g/L) | 0.88 (0.79-0.98) | **0.02** |
| Serum creatinine (µmol/L) | 1.03 (1.01-1.06) | **<0.001** |
| INR^e^ | 27.52 (3.82-198.00) | **0.001** |
| Cystatin C (mg/L) (n=80) | 3.37 (2.19-5.17) | **<0.001** |
| **Falls** | | |
| Frailty (robust/pre-frail/frail according to Fried frailty criteria)^c^ | 2.48 (1.21-5.11) | **0.01** |
| Handgrip strength (kg) | 0.94 (0.89-0.99) | **0.02** |
| PA^d^ ≤4.6º | 4.43 (1.72-11.4) | **0.002** |

^a^Endpoint including hospitalisation, long-term care centre admission, falls, or death; ^b^MELD-Na: Model for end-stage liver disease-sodium; ^c^robust (0 Fried frailty criteria), prefrail (1-2 criteria), and frail (≥3 criteria); ^d^PA: phase angle; ^e^International normalized ratio.

Supplementary Table S5. Added predictive value to MELD-Na when phase angle (PA) was also included in the model for the different endpoints. Evaluated by IDI (integrated discriminating improvement) and NRI (net reclassifitation improvement). Data are expressed as % (95% CI). P values in bold indicate statistical significance.

|  | **IDI (%)** | **p** | **NRI (%)** | **p** |
| --- | --- | --- | --- | --- |
| **Composite endpoint** | 11.6 (4.4-18.8) | **0.002** | 58.5 (17.3-99.7) | **0.005** |
| **Mortality** | 0.3 (-6.6-7.0) | 0.94 | 61.1 (3.0-119.0) | **0.04** |
| **Hospitalisation** | 13.7 (5.3-22.0) | **0.001** | 59.5 (9.7-109.4) | **0.02** |
| **Falls** | 9.6 (3.9-15.3) | **<0.001** | 71.3 (24.9-118.0) | **0.003** |
